# Noise exposure in oil mills

**DOI:** 10.4103/0019-5278.40812

**Published:** 2008-04

**Authors:** G. V. Prasanna Kumar, K. N. Dewangan, Amaresh Sarkar

**Affiliations:** North Eastern Regional Institute of Science and Technology, Nirjuli - 791 109, Itanagar, Arunachal Pradesh, India

**Keywords:** Frequency analysis, hearing loss, noise exposure

## Abstract

**Context:**

Noise of machines in various agro-based industries was found to be the major occupational hazard for the workers of industries. The predominant noise sources need to be identified and the causes of high noise need to be studied to undertake the appropriate measures to reduce the noise level in one of the major agro-based industries, oil mills.

**Aims:**

To identify the predominant noise sources in the workrooms of oil mills. To study the causes of noise in oil mills. To measure the extent of noise exposure of oil mill workers. To examine the response of workers towards noise, so that appropriate measures can be undertaken to minimize the noise exposure.

**Settings and Design:**

A noise survey was conducted in the three renowned oil mills of north-eastern region of India.

**Materials and Methods:**

Information like output capacity, size of power source, maintenance condition of the machines and workroom configurations of the oil mills was collected by personal observations and enquiry with the owner of the mill. Using a Sound Level Meter (SLM) (Model-824, Larson and Davis, USA), equivalent SPL was measured at operator's ear level in the working zone of the workers near each machine of the mills. In order to study the variation of SPL in the workrooms of the oil mill throughout its operation, equivalent SPL was measured at two appropriate locations of working zone of the workers in each mill. For conducting the noise survey, the guidelines of Canadian Centre for Occupational Health and Safety (CCOHS) were followed. Grid points were marked on the floor of the workroom of the oil mill at a spacing of 1 m × 1 m. SPL at grid points were measured at about 1.5 m above the floor. The direction of the SLM was towards the nearby noisy source. To increase accuracy, two replications were taken at each grid point. All the data were recorded for 30 sec. At the end of the experiment, data were downloaded to a personal computer. With the help of utility software of Larson and Davis, USA, equivalent SPL and noise spectrum at each reading was obtained. Noise survey map of equivalent SPL was drawn for each oil mill by drawing contour lines on the sketch of the oil mill between the points of equal SPL. The floor area in the oil mill where SPL exceeded 85 dBA was identified from the noise survey map of each oil mill to determine the causes of high level of noise. Subjective assessment was done during the rest period of workers and it was assessed with personal interview with each worker separately. Demographic information, nature of work, working hours, rest period, experience of working in the mill, degree of noise annoyance, activity interference, and psychological and physiological effects of machine noise on the worker were asked during the interview. These details were noted in a structured form.

**Statistical Analysis Used:**

Nil.

**Results:**

The noise survey conducted in three renowned oil mills of north-eastern region of India revealed that about 26% of the total workers were exposed to noise level of more than 85 dBA. Further, 10% to 30% floor areas of workrooms, where oil expellers are provided have the SPL of more than 85 dBA. The noise in the oil mills was dominated by low frequency noise. The predominant noise sources in the oil mills were seed cleaner and power transmission system to oil expellers. Poor maintenance of machines and use of bamboo stick to prevent the fall of belt from misaligned pulleys were the main reason of high noise. Noise emitted by the electric motor, table ghani and oil expellers in all the oil mills was well within 85 dBA. Subjective response indicated that about 63% of the total workers felt that noise interfered with their conversation. About 16% each were of the opinion that noise interfered in their work and harmed their hearing. About 5% of workers stated that the workroom noise gave them headaches.

**Conclusions:**

The workers engaged in the workrooms of the oil mills are exposed to high noise, which will have detrimental effect on their health. The poor maintenance of drive system was found to be the main reason for high noise level.

## INTRODUCTION

India is one of the major players in the global oilseeds/vegetable oil economy. With the largest area in the world under oilseeds like groundnut, rapeseed-mustard, sesame, safflower and castor, about 27.9 million tons of oil seeds were produced in India during 2005-2006.[1] The production of rapeseed-mustard in India is about 7 million tons, which is about 12% of the world's total rapeseed-mustard production.[2] Oil extraction from rapeseed-mustard is about 2.1 million tons/year.[3] But the rapeseed-mustard oil milling sector in India is a small scale sector and as its activities are not regulated under any legal provisions, it has remained as unorganized sector. These oil mills are using table ghanis and oil expellers for oil extraction and they are situated in the oil seed growing areas, thus providing employment to rural people.

Noise in work environment is the major cause of concern for safety and health of the industrial workers. Since, industrial law in India does not provide any protection to workers from noise pollution,[4] it is considered as a part of routine and the inescapable part of work environment.[5] Due to this, agro-industries do not give much importance to the exposure of workers to high intensity noise and adoption of suitable measures for its control. There are evidences to support this fact of increasing prevalence of high noise levels in the workplaces of various factories in India.[6] Studies carried out by National Institute of Occupational Health (NIOH) showed that the sound pressure levels (SPL) were very high in various industries, ranging from 102-114 dBA in textile industries, 93-103 dBA in pharmaceutical firms, 90-102 dBA in fertilizer plants and 90-119 dBA in oil and natural gas complexes in Bombay High.[7]

Detrimental effect of high level of noise on human health is known for centuries. The exposure to high noise level has both immediate and long-term effects on the workers. Noise disturbs the work, rest, sleep and communication and leads to accidents in industries. It causes physiological, psychological and possibly pathological reactions.[8] The long-term effect noise is hearing loss. Hearing loss due to industrial noise has been studied by many researchers.[9–12] However, hearing loss does not occur in sudden and traumatic manner, but it is imperceptibly slow and painless.[13] At first, the workers are unaware of it, and gradually they notice loss of hearing.[14] International Standard Organization[15] has set out comprehensive information on the risk of loss of hearing in relation to age, duration of exposure and the intensity of noise. Exposure duration of 40 h per week of equivalent noise level of 85 dBA is considered to be safe and noise level above this limit is bound to cause noise induced hearing loss.[16,17]

Since, oil mills are important for the comprehensive development of rural economy of India, it is very essential to protect the workers from ill-effects of noise. However, the literature available on the extent of exposure of workers to high noise level in Indian oil mills is limited. Keeping these factors in view, an investigation was conducted to identify the predominant noise sources in the workrooms of oil mills, causes of high noise and the extent of noise exposure of oil mill workers. Attempts were also made to examine the response of workers towards noise, so that appropriate measures can be undertaken to minimize the noise exposure.

## MATERIALS AND METHODS

### Description of oil mill

The study was conducted in three renowned oil mills located in the north-eastern region of India. These mills were involved in expelling oil from rapeseed-mustard for the last 15-18 years. All the selected oil mills were established by small and medium entrepreneurs. The various processing operations performed in the workrooms of oil mill were: seed cleaning, crushing of seeds, expelling of oil and filtration. Seed cleaning was done by a seed-cleaning machine in oil mill 1 whereas in oil mills 2 and 3, it was accomplished manually. For crushing of seeds, table ghanis were used. Table ghani is the traditional oilseed crushing unit used in India and FAO[18] has the details of its construction and working. Oil was expelled from the seeds using screw type oil expeller. In oil mill 1, after cleaning, seeds were directly fed to the expeller, whereas seeds were initially crushed in table ghanis and then fed to the expeller in oil mills 2 and 3. Oil mills 2 and 3 used 24 table ghanis and 2 oil expellers. Table ghani being a batch type machine, the seeds were loaded and crushed material is unloaded manually. For feeding the seeds to the expellers, each expeller is provided with a bucket elevator. The expelled oil is then filtered in filter press.

The source of power to all the machines in the oil mill was a 3-phase induction motor. From motor shaft, power was taken to a long shaft. The long shaft drives all table ghanis through flat belt drive and gear drive. The seed cleaner, oil expellers and bucket elevators receive the power from the long shaft through flat belt drive. In oil expelling and filtering room of oil mill 2, each oil expeller along with bucket elevator was provided with an electric motor individually.

### Experimental details

The study was conducted during March-April month, which was the peak period of oil milling in the region. The working hours of the oil mill during peak period of milling was from 7 am to 12 noon whereas during lean period, it was operated for 2-3 hours depending on the quantity of seeds available for oil expelling. The noise data were collected between 8 and 11 am. The average temperature and relative humidity during the period of investigation in the workrooms of the mill was respectively 25 ± 2°C and 74 ± 3%. A Sound Level Meter (SLM) (Model-824, Larson and Davis, USA) was used to record noise level. SLM was set for recording in slow mode in weighting scale “A”. The SLM was calibrated before the start of the study.

### Collection of data and analysis

The written approval was obtained from the owner of each oil mill for conducting noise survey. Information like, output capacity, size of power source, maintenance condition of the machines and workroom configurations of the oil mills were collected by personal observations and enquiry with the owner of the mill.

The equivalent SPL was measured at operator's ear level in the working zone of the workers near each machine of the mills. In order to study the variation of SPL in the workrooms of the oil mill throughout its operation, equivalent SPL was measured at two appropriate locations of working zone of the workers in each mill.

For conducting the noise survey, the guidelines of Canadian Centre for Occupational Health and Safety (CCOHS)[19] were followed. Grid points were marked on the floor of the workroom of the oil mill at a spacing of 1 m × 1 m. The (0, 0) coordinate was appropriately taken on the extreme left hand corner of the mill. In case the machines in the mill interrupted with the grid points, grid points were marked on either sides of the machine. The coordinates of the rectangular area under each machine were noted down to mark the location of machines in the mill. SPL at grid points were measured at about 1.5 m above the floor. The direction of the SLM was towards the nearby noisy source. To increase accuracy, two replications were taken at each grid point. All the data were recorded for 30 sec.

At the end of experiment, data were downloaded to a personal computer. With the help of utility software of Larson and Davis, USA, equivalent SPL and noise spectrum at each reading was obtained. Noise survey map of equivalent SPL was drawn for each oil mill by drawing contour lines on the sketch of the oil mill between the points of equal SPL. The floor area in the oil mill where SPL exceeded 85 dBA was identified from the noise survey map of each oil mill to determine the causes of high level of noise.

### Assessment of subjective response

The oil mills employ large number of workers for various operations like, cleaning seeds, feeding seeds to table ghanis and collecting cakes from oil expellers. All the workers engaged in the workroom were considered for the assessment of subjective response. Prior to noise survey, a meeting was conducted in each oil mill with the workers. The purpose of noise survey and its application were convinced and their cooperation was sought in the meeting. Subjective assessment was done during the rest period and it was assessed with personal interview with each worker separately. Demographic information, nature of work, working hours, rest period, experience of working in mill, degree of noise annoyance, activity interference, and psychological and physiological effects of machine noise on the worker were asked during the interview. These details were noted in a structured form.

## RESULTS AND DISCUSSION

**Figure 1:**
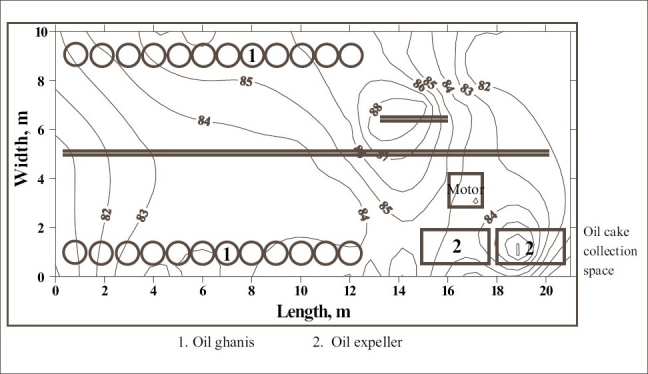
Noise survey map of oil expelling room of oil mill 2

**Figure 2:**
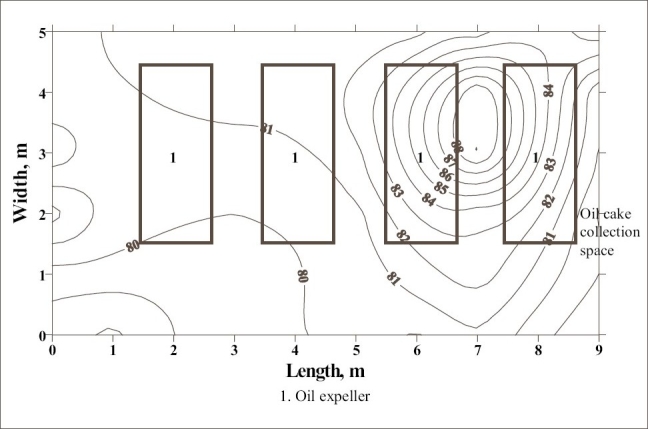
Noise survey map of oil expelling and filtering room of oil mill 2

**Figure 3:**
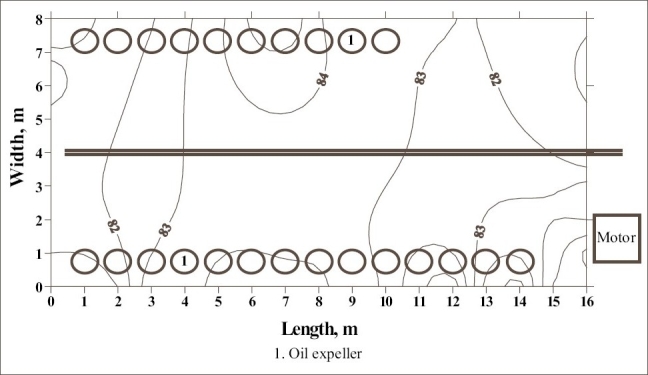
Noise survey map of oil expelling room of oil mill 3

The details of the oil mill, viz., number of workrooms, machines in each workroom, size of the electric motor, floor area of the mill, floor area under the machines and maintenance level of the machines are presented in Table 1. The arrangement of the machines in the workrooms of the oil mill are presented in the noise survey maps [Figures 1-3].

**Table 1:** Details of oil mills considered for the study

Oil mill	Workroom	Machines	Size of electric motor, kW	Floor dimension, m × m	Floor area under the machines, m × m	Remarks on maintenance
1	Seed cleaning and oil expelling room	Seed cleaner-1	22.3	28 × 15	2.5 × 1.5	Poor
		Table ghani-24			16 × 10	-
		Oil expeller-2			6 × 4	Good
2	Seed feeding room	-	-	15 × 4	-	-
	Oil expelling room	Table ghani-24	37.1	23 × 12	20 × 10	Moderate
		Oil expeller-2				Moderate
	Oil expelling and filtering room	Oil expeller-4	89*	15 × 8	9 × 5	Moderate
		Press filter-1				-
3	Table ghani room	Table ghani-24	44.5	20 × 9.5	16 × 8	Good
	Oil expelling room	Oil expeller-2	29.7	13 × 9.5	6 × 4	Moderate

* Each expeller in the mill is operated by individual electric motor of 22.25 kW

### Demographic characteristics of the study population

The workers engaged in the oil mills were in the age group of 26 to 56 years. The mean age of all the workers was about 42 years. They had 5 to 15 years of experience of working in the oil mill. About 64% of the workers were female. Due to seasonal availability of mustard seeds, the oil mills were operated for about 210 to 220 days in a year and for about 2 to 5 hours per day. Therefore, the workers of the oil mill work in the rice mill owned by the same entrepreneur after their engagement in the oil mill. On an average they work for 56 hours a week (8 hours per day, 7 days a week) during peak period and 48 hours a week (8 hours per day, 6 days a week) during lean period. Male workers were employed mainly for the heavy task, like feeding seeds to the seed cleaner and near the oil expellers. Female workers were employed for loading and unloading of table ghanis, collection of oil cake from the expeller and cleaning of the spill over material in the mill.

### Noise in workrooms

#### Noise at operator's ear level

The values of equivalent SPL at operator's ear level at various locations of the workroom of oil mills is presented in Table 2. It reveals that among all the machines in the oil mills, seed cleaner of oil mill 1 produced the highest noise and it is in the range of 85.5 to 89.5 dBA. At this location five workers are engaged [Table 3], which is about 45% of total workers engaged in the workroom of this mill. Workers engaged at this location are more susceptible to excessive noise than those engaged near the oil expeller of the same mill. In general, the noise emitted from most of the table ghani and oil expeller was within acceptable limits except for few table ghanis of oil mill 2 and one oil expeller of oil mills 2 and 3. Noise near table ghanis and oil expeller of oil mill 2 had the effect on the workers as they work near these machines. Oil expeller of oil mill 3 had the high noise near the power transmission side and workers rarely work at this location.

**Table 2:** Equivalent SPL at operator's ear level at various locations of oil mills

Oil mill	Workroom	Machine	Locations	L_eq_ (dBA)
1	Seed cleaning and oil expelling room	Seed cleaner	Feed side	87.1
			Clean seed outlet side	85.5
			Dust outlet side	89.5
		Oil expeller	Feed hopper	76.3
			Front side	78.7
			Power transmission system to the expeller	79.7
			Oil cake outlet	77.9
2	Seed feeding room	-	Seed feeding pit	74.0
	Oil expelling room	Table ghani	Seed feeding area of 12^th^ table ghani in the front row	84.6
		Table ghani	Seed feeding area of 9^th^ table ghani in the rear row	85.6
		Oil expeller	Oil cake outlet of oil expeller	80.6
	Oil expelling and filtering room	Oil expeller	Oil cake outlet of 1^st^ oil expeller	81.3
		Oil expeller	Oil cake outlet of 2^nd^ oil expeller	82.0
		Oil expeller	Oil cake outlet of 3^rd^ oil expeller	87.4
		Oil expeller	Oil cake outlet of 4^th^ oil expeller	82.5
3	Table ghani room	Table ghani	Seed feeding area of 14^th^ table ghani in the front row	84.5
		Table ghani	Seed feeding area of 8^th^ table ghani in the rear row	84.0
	Oil expelling room	Oil expeller	Feed hopper	78.1
		Oil expeller	Front side	77.6
		Oil expeller	Power transmission system to the expeller	85.6
		Oil expeller	Oil cake outlet	77.6

**Table 3:** The distribution of the workers in the workroom of oil mill

Oil mill	Seed feeding	Seed cleaner	Table ghani	Oil expeller	Total
1	-	5	-	6	11
2	4	-	6	12	22
3	-	-	6	4	10

The noise emitted during the operation of seed cleaner was high due to the oscillations of sieves and use of crank and pitman mechanism to provide the oscillating motion to sieves. The poor maintenance condition of the seed cleaner is also another reason for high noise level. As table ghanis and oil expellers operate at low speed and have smooth rotating components, they emitted low noise level. However, the power transmission system to the oil expellers produced high noise level in the workroom. In general, the horizontal and vertical distance between the shaft supplying power to the expeller and the shaft driving the bucket elevator of the expeller was more than 4 and 2 m respectively. In order to prevent the frequent falling of the flat belt of the power transmission system from misaligned pulleys, each oil mill provided bamboo supports on either side of the belt. Rubbing of the belt to the bamboo supports produced very high noise level. The SPL of power transmission system to the table ghanis was within permissible limit as the belt drive was not provided with any supports.

The distribution of the total subjects by equivalent continuous noise level during the operation of the oil mill is presented in Table 4. This reveals that, a total of 11 laborers accounting for about 26% of the total laborers engaged in the workrooms of all the oil mills were exposed to noise level of more than 85 dBA. This group of workers included five women (12% of total labourers).

**Table 4:** Distribution of the total subjects by equivalent continuous noise level during the operation of oil mill

L_eq_ in dBA	Oil mill 1	Oil mill 2	Oil mill 3	Total
<80	6	4	4	14
80-85	-	12	6	18
85-90	5	6	-	11
Total	11	22	10	43

### Noise survey of workrooms

The noise survey map of the oil expelling room of the oil mill 2 is shown in Figure 1. It indicates that the SPL in the workroom varied from 80 to 88 dBA and about 30% of the floor area of the workroom had SPL of more than 85 dBA. The high noise level is concentrated in the floor area nearby power transmission system. The floor area under high noise included the working zone of two workers engaged near nine table ghanis in the rear row of ghanis of the mill. [Figure 2] shows the distribution of SPL in the oil expelling and filtering room of the same oil mill. The SPL higher than 85 dBA was found to be concentrated between 3^rd^ and 4^th^ oil expeller in about 10% of the total floor area of the workroom. In this workroom, each oil expeller along with bucket elevator is provided with separate electric motor. The pulleys of 3^rd^ and 4^th^ oil expeller were out of alignment and hence, bamboo strips have been provided as supports to prevent the falling of belt out of the pulley. This is the cause of excessive noise in this location of the workroom. This high noise had effect on five workers engaged in cleaning, collection and recirculation of oil cakes in the 3^rd^ oil expeller. The noise survey map of equivalent SPL of the oil expelling room of oil mill 3 is presented in Figure 3. The SPL in the workroom was found to be within permissible limits. This is due to good maintenance of the machines of the mill.

**Figure 4:**
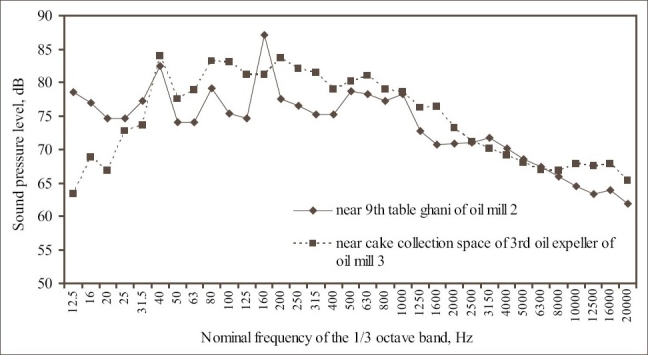
Frequency analysis of SPL at the working zone of excessive noise exposure in oil mills 2 and 3

The frequency analyses of noise in 1/3^rd^ octave band at the 9^th^ table ghani of oil mill 2 and near cake collection space of 3^rd^ oil expeller of oil mill 3 are presented in the Figure 4. The noise at table ghani showed flat trend with two dominant noises at 40 and 160 Hz. At this location, the SPL decreased considerably after 1000 Hz frequency. The SPL at oil expeller was more than 76 dB in the frequency range from 40 to 1600 Hz, with the most dominant noise at 40 Hz.

### Variation in SPL during operation of mill

**Figure 5:**
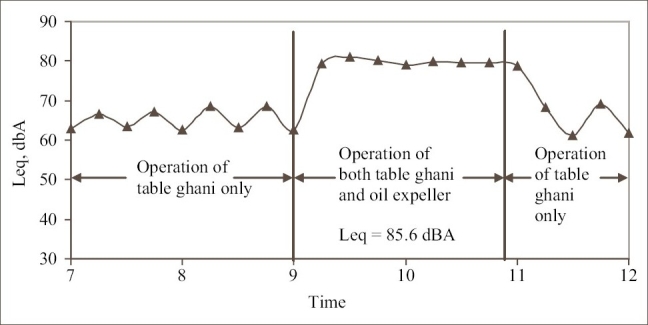
Typical daily noise exposure in the oil expelling room of oil mill 2 near the seed feeding area of 9^th^ table ghani

The workers were exposed to noise of continuous type in the oil mills. The overall noise level in the oil mills ranged between 78 and 92 dBA. As an example, the variation in daily noise exposure of the worker in the oil expelling room of oil mill 2 working near seed feeding area of 9^th^ table ghani is presented in Figure 5. Noise exposure at this location is presented because this falls under the zone of excessive noise exposure. At every 20 min interval, the table ghanis are loaded and unloaded. As the drive to the table ghani is disconnected during unloading and loading operation, the SPL varied during the period from 7 to 9 am. The oil expeller is operated only during the period from 9 to 11 am. depending on the seed availability. The noise emitted from the flat belt drive to oil expeller resulted in the increase of SPL in the workroom. The SPL once again reduced when the expeller was stopped.

### Subjective response of the noise

The results of the subjective response to noise in the oil mills illustrated the risk of hearing damage among the workers particularly working in the predominant noise zone. About 63% workers said that the noise interfered with their conversation. This is found to depend on the distance from the peak noise source. Most of the workers who had difficulty in conversation were working near oil expeller. Difficulty in conversation may be due to the fact that the frequency of human noise is in the range of 300 to 700 Hz and the oil expeller emitted noise of more than 80 dBA in this frequency range. Therefore, it is difficult to distinguish between machine noise and human noise. About 16% were of the opinion that noise interfered in their work. However, physiological effects were considered less significant. About 5% of workers stated that the workroom noise gave them headaches. About 16% workers were of the opinion that they had little problem of hearing. The workers who had headache and problem of hearing were of more than 45 years of age.

## CONCLUSIONS

The noise survey conducted in three renowned oil mills of north-eastern region of India revealed that about 26% of the total workers were exposed to noise level of more than 85 dBA. Further, 10 to 30% floor areas of workrooms, where oil expellers are provided have the SPL of more than 85 dBA. The noise in the oil mills was dominated by low frequency noise. The predominant noise sources in the oil mills were seed cleaner and power transmission system to oil expellers. Poor maintenance of machines and use of bamboo stick to prevent the fall of belt from misaligned pulleys were main reason of low noise. Noise emitted by the electric motor, table ghani and oil expellers in all the oil mills was well within 85 dBA. Subjective response indicated that about 63% of the total workers felt that noise interfered with their conversation. About 16% each were of the opinion that noise interfered in their work and harmed their hearing. About 5% of workers stated that the workroom noise gave them headaches.

## References

[1] Hegde DM (2007). Oilseeds: Increasing production area. The Hindu Survey of Indian Agriculture.

[2] Damodharan T, Hegde DM (2005). Oilseed situation: A statistical compendium 2005.

[3] Pahariya NC, Mukherjee C (2007). Commodity revenue management: India's rapeseed/mustard oil sector.

[4] Directorate General; Factory Advice Service and Labour Institutes Ministry of Labour (2003). Existing setup of occupational safety and health in the workplace, Chapter 1.

[5] Bhat S (2003). A few reflections of noise pollution: Issues and concerns in urban India. http://www.indiatogether.org/2003/nov/law-noise.htm.

[6] Melamed S, Fried Y, Froom P (2001). The interactive effect of chronic exposure to noise and job complexity on changes in blood pressure and job satisfaction: A longitudinal study of industrial employees. J Occup Health Psychol.

[7] National Institute of Occupational Health [Homepage on the Internet] Generation of Database on Occupational Diseases (Achievements). http://www.icmr.nic.in/000004/achievements.htm.

[8] Raju S (2003). Noise pollution and automobiles. Proceedings of the Symposium of International Automobile Technology.

[9] C'elik O, Yalc'ın S', Ozturk A (1998). Hearing parameters in noise exposed industrial workers. Auris Nasus Larynx.

[10] Ahmed HO, Dennis JH, Badran O, Ismail M, Ballal SG, Ashoor A (2001). Occupational noise exposure and hearing loss of workers in two plants in eastern Saudi Arabia. Ann Occup Hyg.

[11] Eleftheriou PC (2002). Industrial noise and its effects on human hearing. Appl Acoust.

[12] Miyakita T, Ueda A, Futatsuka M, Inaoka T, Nagano M, Koyama W (2004). Noise exposure and hearing conservation for farmers of rural Japanese communities. J Sound Vibr.

[13] Sanders MS, McCormick EJ (1992). Human factors in engineering and design.

[14] Grandjean E (1988). Fitting the task to the man.

[15] ISO-1999 (1990). Acoustics-determination of noise exposure in workplace and evaluation of the auditory damage due to noise.

[16] National Institute of Occupational Safety and Health (1996). Criteria for a recommended standard occupational noise exposure. NIOSH - Education and Information Division, Division of Biomedical and Behavioral Science, Draft Document.

[17] Rick N (2004). Noise exposure standards around the world [monograph on the Internet]. http://staff.Washington.edu/meitzel/standards.htm.

[18] Food and Agriculture Organization (FAO) of United Nations Ghani-Atractional method of oil processing in India [monograph on the Internet]. http://www.fao.org/DOCREP/T4660T/t4660t0b.htm.

[19] Canadian Centre for Occupational Health and Safety (CCOHS) Noise - Measurement of workplace noise [monograph on the Internet]. http://www.ccohs.ca/oshanswers/phys_agents/noise_measurement.html.

